# Remote Interpreting in Immigration Tribunals

**DOI:** 10.1007/s11196-022-09908-3

**Published:** 2022-06-03

**Authors:** Tatiana Grieshofer

**Affiliations:** Birmingham City University, Birmingham, UK

**Keywords:** Immigration appeals tribunal, Remote interpreting, Online hearings, Role of interpreters, Communicative practices

## Abstract

As part of the response to the COVID-19 pandemic, many jurisdictions across the world introduced remote hearings as an alternative way of continuing to offer access to courts. This practice-based article discusses the report prepared by the author for a judicial review case which revolved around the claim that in immigration settings the quality of interpreting conducted in fully online hearings is inferior to interpreting in face-to-face hearings. In the absence of pre-existing research comparing the impact of the physical and fully online settings on interpreting, the author’s expert witness report explored linguistic principles governing conversation and turn-taking management, power relations and narrativisation and discursive practices in online and physical settings to illustrate communicative advantages and disadvantages of each environment. The article draws on the investigations conducted for the expert witness report and pursues the following aims: (1) reflect on the role of linguistic expertise required for the case; (2) detail the conclusions drawn and recommendations endorsed in the report; (3) discuss the importance of effective communication in immigration settings; (4) challenge common misconceptions in relation to how narratives are elicited, shared and perceived; (5) explore safeguarding strategies for enhancing discursive practices in fully remote hearings in order to improve non-native speakers’ access to justice.

## Introduction

The article draws on my expert witness report for a judicial review case, which raised questions about communication practices in interpreter-mediated remote immigration hearings where all participants are present online. As the COVID-19 pandemic hit the world and it became instrumental to introduce restrictions on face-to-face contact in private and professional settings, the courts and tribunal services across different jurisdictions had to adapt to ensure that the cases could be heard in compliance with the local public health measures. As a result, some of the justice services had to be digitised and many court hearings had to be offered in a remote mode, i.e. via telephone or online. Different countries were at different stages of preparedness for the introduction of remote hearings.^1^ For instance, the legal system of England and Wales was amid the ongoing digitisation reform of the HMCTS (Her Majesty’s Courts and Tribunals Service) at the beginning of 2019; testing video technology was part of the reform (e.g. a pilot on remote hearings in the tax tribunal)^2^ and the pandemic thus accelerated the introduction of remote hearings more widely across the courts and tribunals. Elsewhere, the ongoing pandemic prompted the development of digitisation solutions and expedited the establishment of support strategies for court users and practitioners. In Brasil, video guidance has been issued for lawyers on instructing clients and conducting virtual proceedings. Italy has initiated the introduction of smart courts with remote support, chatbots, access to online files and a contract tool with further plans for incorporating AI elements for drafting documents and tracking the progress of proceedings.^3^

The rapid introduction of remote hearings led to the need for the legal systems across the world to define the suitability of the remote mode of hearings for different types of cases. In England and Wales, for instance, the appropriateness of the remote mode for hearings is determined by the judiciary on a case-by-case basis. The legislation around the offer of remote hearings is currently being shaped as more cases enter case law and start framing the legal stance to the suitability of remote hearings in diverse circumstances. For instance, a law firm in California successfully opposed the remote hearing format for an antitrust case on the basis that it would be “fundamentally unfair”.^4^ Similarly, the Supreme Judicial Court in Massachusetts is currently soliciting briefs on whether online hearings can be held in cases about terminating parental rights if one of the parents objects to their case being presented remotely.^5^ Interpreter-mediated remote hearings, which are the focus of this article, present an equally challenging set of circumstances in relation to language and communication. Although many countries across the world have made the decision that remote hearings should be a standard part of the justice provision post-pandemic, the research on comparing face-to-face hearings to remote hearings is largely missing at the stage of writing.

This practice-based article draws on the expert witness report I was commissioned to write in 2021. The report was for a judicial review case in relation to an immigration appeal case in a common law country (the case details will remain anonymous due to the vulnerability of immigration appellants and the theoretical focus of the expert report required for the case). The appeal case was initiated by the appellant’s lawyers upon the refusal of the appellant’s refugee status. As soon as the immigration appeal tribunal scheduled the appeal hearing to be conducted online, the appellant’s lawyers submitted an application for the judicial review to be held ahead of the appeal hearing. The judicial review application was broadly brough on the basis that (1) there was no legal ground for scheduling the appeal hearing online and (2) the pre-emptive concern that online settings would be disadvantageous for the appellant due to additional pressures put on interpreters, who are often underqualified and unregulated in the given jurisdiction, potentially leading to the appellant’s credibility to be disproportionately undermined. My report was prepared for the defendants, the immigration appeals tribunal, in response to the expert witness report commissioned by the appellant’s legal team. The report was accepted by the judge presiding over the judicial review case, but the case was eventually settled and the appellant was offered an in-person court date as the COVID-19 restrictions were starting to be eased. Despite the fact that there was no decision taken, the case brought to light several crucial aspects central to spoken interaction in legal settings: the role of interpreters, the regulation of interpreters, communicative practices in face-to-face and remote hearings, misconceptions around communication and perceptions of credibility.

The key part of the appellant’s case was the expert witness report from an academic in translation studies; the report detailed a wide range of aspects: interpreting in the context of immigration tribunals, the poor local standards of interpreter provision, the lack of accreditation or training programmes for interpreters and the adverse impact this has on the quality of interpreting, negative effect of online settings on the quality of interpreting, and misguided perceptions of applicants appearing online. The expert report presented by the appellant’s team was partially based on the inaccurate interpretation of linguistic principles, which led to the conclusion that online interpreting is likely to be more problematic than face-to-face interpreting due to additional stress, technical issues, toxic sound, poor lip synchronisation, overflow of visual information, and the need to rely on notes and memory for consecutive interpreting if simultaneous interpreting is not possible.

The letter of instructions from the defendants’ legal team asked me to: provide a response to the existing expert witness report; assess whether it is generally possible to have an effective interpreter-mediated remote hearing; express an opinion on whether conducting a remote hearing with several participants could “cause a significant loss of comprehension over and above the linguistic obstacles which might be expected in a traditional physical hearing format”; establish if toxic noise or occasional background activities would have a detrimental effect on the efficient conduct of a remote hearing under typical working conditions; assess if it is possible to address any potential challenges for interpreters by following specific safeguarding procedures; and evaluate the existing safeguarding procedures already adopted by the tribunal. The overlap in the points illustrates that there was some confusion as to what exactly remote interpreting involves. In sum, the misconceptions present in the expert report supporting the appellant’s claim and some confusion over linguistic principles governing effective communication in the letter of instructions illustrate the need for exploring aspects the article focuses on: (1) supporting courts and tribunals in establishing communication practices, (2) reflecting on the role of linguistic expertise in legal settings, and (3) raising the awareness of legal professionals in the importance of language and communication for access to justice.

The case was unusual in the sense that there was no data analysis involved as the appellant’s claims related to potential issues which could be hypothetically more frequent in online hearings. Although linguists usually contribute to court cases by providing data analysis related to, for instance, authorship analysis, suspect profiling, the veracity of disputed statements [16], the generic nature of the expert witness report discussed here resembles the characteristics of the anthropological expertise required in immigration tribunal settings [30] or linguistic expertise required for trademark and other proprietary litigation [13]. In order to reflect on the potential communicative challenges involving an interpreter at a future remote hearing, my expert witness report drew on the following foundations: (1) the existing research on courtroom discourse and challenges for interpreters in legal settings; (2) my prior research on discursive practices embedded in court and tribunal procedures [31, 32, 57] based on court observations and grounded in socio-legal studies, ethnography of communication, Conversation Analysis, Critical Discourse Analysis and narrative analysis methods; (3) the investigations I conducted to explore the existing communicative practices during remote immigration tribunal hearings, resulting in a list of recommendations (see below). As a result, the report explored the impact of different interaction patterns, power relations and physical surroundings on potential communicative challenges as well as proposed specific recommendations as to how communication can be safeguarded in the remote environment. The discussion in this practice-based article is thus relevant not only in the circumstances of the given case, but also for managing communication processes across a wide range of (interpreter-mediated) remote hearings.

The following sections establish the linguistic context of immigration tribunal hearings and narrativisation practices within immigration settings before discussing the role of interpreters and challenges of interpreter-mediated communication in legal settings. The final sections then explore communication practices in remote hearings, challenge misconceptions about credibility assessment, and provide recommendations on how to manage communication in remote hearings and keep record of the interpreting process.

## Narrativisation in Immigration Settings

The appellant’s story is central to the immigration tribunal cases and the different reiterations of the story are tested throughout the proceedings. The main opportunities for the narrative to be presented occur during (1) the initial (screening) interview between the appellant and a case worker, (2) a witness statement or a form (e.g. a questionnaire in Ireland or statement of evidence forms in the UK), (3) a more substantive interview with another case worker or official and, sometimes, (4) an interview with a doctor or another expert who may be preparing a report, and, potentially, (5) the testimony (examination-in-chief and cross-examination) at an appeal hearing in case the application is rejected and the appellant decides to appeal the decision [29]. So, at the minimum, there is an initial presentation of the story through an (interpreted) question/answer interaction mode, followed by a detailed presentation of the narrative in a written mode (ideally, with the support of a lawyer and potentially an interpreter), and culminated with another detailed (interpreted) question/answer interaction mode but in a more adversarial context, during which asylum seekers are constrained to answering specific questions. Such narrativisation context with several reiterations, mostly fragmented through a dialogic interaction pattern and non-linear approach to the events, is characterised by ‘narrative disjunction’ [16: 111, 57, 32], which is common in legal settings but unfamiliar in lay settings.

The fragmentary nature of elicited micro narratives is further complicated by the fact that the stories are communicated through an interpreter and it is not uncommon for the interpreters to vary across the application stages, which potentially introduces an additional factor that can disrupt the coherence threads in between the micro narratives. An important step in the proceedings is the assessment of the credibility of the appellant’s story, which is established by evaluating internal consistency (consistency among the different reiterations of the story), external consistency (consistency between the story and evidence) and general plausibility [56, 61]. The credibility assessment thus relies on such inherently linguistic aspects as (1) accurate transposition of the content and linguistic framing of the story from the appellant’s mother tongue to the target language and (2) the projection of the appellant’s authentic voice (though this is often complicated by the eliciting strategies [32]). However, the appellant’s narratives are often created and shaped in the context of ‘the culture of disbelief’ characteristic of immigration tribunals with prevailing “themes of authority, distrust, inconsistency, chaos and otherness” [1]. As a result, multiple linguistic, discursive, socio-cultural, attitudinal, emotional, systemic and procedural aspects impact how the narratives are presented, argued and perceived [1, 32]. The narrativisation journey thus involves the interpretation of not only linguistic aspects but also socio-cultural characteristics embedded in the appellant’s story and its different reiterations throughout the application process.

The tribunal members ultimately assess the social aspects of credibility based on their pre-established cultural norms [62]. The applicant’s narratives thus need to be compatible with the cultural norms and values of the host community while also being conceptualised within the legal framework of the relevant immigration legislation [15]. To achieve both aims, lawyers tend to take an authorial role over their client’s image [15] as they negotiate the meaning of their clients’ narratives through linguacultural, linguistic and discursive lenses [48]. The tensions between the authenticity of the applicant’s voice, the legal framework their stories are constructed in and the host community’s socio-cultural norms create a challenging context for interpreting.

Equally, the culture and overarching narrative of the justice system also plays a role in complicating the context for applicants and interpreters. Much of the research on immigration tribunals highlights that the adjudicative environment or the general atmosphere of immigration tribunals presents multiple challenges for the participants due to the chaotic nature of interactions and imbalanced power relations [1, 3, 10, 28, 29, 51]. The systemic issues include often unrealistic expectations and requirements in relation to the applicants’ narratives: full engagement with institutions and timely disclosure [3, 29]; compliance with formal genre characteristics and alignment with procedural stages (e.g. sufficient level of detail appropriate for the application stage); meeting an imaginary threshold for authenticity and emotional expressiveness of the narrative [3, 29, 51]. The eliciting processes, tribunal procedures and the context in which interactions happen are not conducive to creating a physical and communicative space in which an emotional account can be shared [51]. As a result, the process often leaves appellants feeling imposed on and discredited, leading to a potential disengagement and decreased participation [27].

It is equally important to remember that interpreting is a complex task, especially in the context of immigration and legal settings as these are characterised by coercive interactions, during which language is used to manipulate participants into specific responses, reinterpret the framing of narratives and discredit the credibility of the participants or their presentation of events [26: 112]. Attaining an accurate representation of the original message in the interpreted text, especially in terms of not only factual content but also pragmatic connotations, seems like an almost unrealistic task [8]. The act of interpreting may thus alter the pragmatic force behind the questions as cross-linguistic equivalents can be difficult to find and if the interpreter is not aware of the importance of pragmatics, the chances that the intended meaning can be skewed are higher [9, 50]. Furthermore, the interpreter’s conscious and unconscious biases (e.g. personal beliefs, cultural paradigms, or even prejudices as to the authenticity of the client’s claim or the client’s role in the proceedings, i.e. interpreting for a witness vs. defendant) can also impact the outcome of interpreting and, subsequently, the effect the interpreted message would have on the audience [9]. Equally, the fact that interpreting slows the questioning process often creates complications for legal professionals [50] and creates an environment in which interpreters feel under even more pressure to perform the task. The quality of the evidence collected from the adjudicator’s interaction with the appellant often depends on the interpreter, but this aspect is rarely acknowledged when the internal credibility of the appellant’s narrative is judged.

What is also directly linked to the role of interpreters is the discursive and narrativisation practices in which the interpreting is conducted, i.e. the practices of eliciting narratives and keeping records of different reiterations of the macro narrative. The only constant in the process is the appellant as case workers or adjudicators (and interpreters) often change between the different phases of the proceedings, so there is little continuity among the interlocutors throughout the process in many jurisdictions (e.g. Ireland, England and Wales, France, Belgium). Moreover, each reiteration of the narrative is produced under different circumstances: an informal interview room or booth; a lawyer’s office; another interview room in more formal settings; and, potentially, a quasi-legal environment of immigration appeals tribunals, which share many physical and interactional characteristics with courtroom settings. The different stages are characterised by different legal contexts and inherent moral judgements [18] as well as interactional dynamics [10, 25] and procedural goals. The aim for eliciting the stories and the focus of the stories thus constantly shift. As a result, the appellants have minimal control over the topics (these are defined by interviewer or adjudicators) or the final framing of their narratives (defined by an interpreter and recorded by an interviewer). Furthermore, the appellants’ disempowered institutional position does not encourage them to take interactional space and raise communication-related issues. The tribunal members thus assess credibility through the distorted versions of stories, created via the lenses of several interviewers and interpreters.

From the practical viewpoint, many of these distortions are unavoidable. But what is crucial is for the legal professionals, adjudicators and tribunal services to be aware of these distortions. Court or tribunal procedures need to enable efficient communication management and introduce safeguarding measures to keep an accurate record of original interactions [44]. At the moment, many jurisdictions do not audio/video record the interviews or tribunal proceedings (e.g. US, France, Ireland), which means that often the only record that exists is the notes taken by case workers and adjudicators. Furthermore, the fact that the case workers are the ones conducting the interviews and at the same time creating the only record of the interview reduces both of their abilities, i.e. to engage with the appellants and create a reliable record of the interview [25]. Given that the interviewers fulfil multiple roles and their notes are based on the interpreter-mediated communication or communication with a non-native speaker (i.e. the applicant), the usability of the notes for assessing internal credibility should be brought under question and further researched. What was of particular significance for the expert witness report discussed here is the absence of the interaction in a foreign language in any record. For instance, Berk-Seligson [9: 195] notes that even in situations in which there is transcriber present to produce a verbatim record of the bilingual proceedings, the transcript only includes English [cf 44]. Furthermore, it is important to note that verbatim transcripts of court, tribunal or police interactions, are not an accurate representation of the original interactions [19, 24, 49, 60], let alone if one of the languages is not captured in any form. The fact that interviews and tribunal proceedings are not audio/video recorded illustrates that the justice system puts unrealistic expectations on interpreters without offering many assurances to appellants. The incognisance of the inherent linguistic and socio-cultural complexities within the interpreting process in immigration settings constitutes a critical barrier for access to justice for non-native speakers [9].

## Standard of Interpreting

The quality of interpreting is important for access to justice, yet there is often not sufficient recognition of challenges involved in the interpreting process: the stakes for appellants are high, the legal professionals’ expectations of interpreters are significant and the working context for interpreters is challenging; yet, the presence of interpreters is “tolerated rather than welcomed” [35] and their training needs and accreditation standards are not always suitably established [9]. Since the judicial review case reported here was related to the poor standards of interpreter qualifications accepted in legal settings, it is important to clarify the practical considerations related to interpreting standards and how interpreted issues are reflected in the appeals proceedings.

The general consensus is that the recommended entry requirements into the profession should include (1) bilingualism, (2) achieving a high threshold level in a certification exam that tests technical interpreting skills and the knowledge of a specialised lexical domain and, finally, (3) the willingness to participate in continuous professional development [9]. The effectiveness of specialised training has been shown to help increase the accuracy of interpretation [40]. Yet, the standards for regulating the practice of interpreters differ widely across jurisdictions. In Europe, there is a strong tradition of interpreting and translation studies with clear paths for accreditation for court-certified interpreters [4, 9] and more recent attempts to create an EU-wide professional register of interpreters, based on objectively measurable and reliable criteria [12, 41]. In the Czech Republic, for instance, court-certified interpreters need to have a master’s degree, show evidence of the expert knowledge of the language, complete a year-long course for legal interpreters and translators, and demonstrate the relevant 5-year experience.^6^ Elsewhere, the situation is more varied; in the US, there is a federal certification programme and a wide range of training opportunities for existing interpreters [9: 210]. By contrast, in Ireland there is currently no certification system for accrediting legal interpreters or the provision of interpreting and translation services across public bodies [46]. Although this should not be a standard situation, on occasions when there is an insufficient number of interpreters or when there are no certified interpreters for a specific language, it is understandable that the non-accredited interpreters need to be recruited. To address potential complications in such situations, Berk-Seligson [9: 243] suggests offering different levels of certification guided by the experience and training achieved: providing clarity about the interpreter’s training can help manage legal professionals’ expectations.

As highlighted above, there can be a wide range of issues with the quality of interpreting. Interpreters, however, rarely undergo the same degree of scrutiny other expert witnesses are subjected to (Ahmad 2007 qtd. in [33]) as their qualifications and expertise do not tend to be challenged in legal proceedings. When discussing cases in which interpreting was mentioned as one of the grounds for appeals, Berk-Seligson [9: ch. 9] reports on the following reasons cited in judgements: inaccurate interpreting, interpreter’s bias or conflict of interest, and issues with interpreting procedures or techniques. The lower court decisions tend to be upheld in the appeals, mainly because of the absence of evidence of problematic interpreting since the testimony in a foreign language is usually not recorded (see the section above on the lack of requirement for recording interviews and the reliance of the process on monolingual notes/transcripts). There are thus only two situations in which the official record can reflect issues with interpreting: either there is a concern raised on record by one of the participants or the record of the appellant’s responses shows signs of incomprehensible interaction. In relation to the first condition, concerns are mostly voiced by the appellant’s lawyer and only rarely by the appellants themselves simply because there are several important aspects linked to voicing concerns: fluency in both languages to be in the position to notice linguistic problems; institutional role which allows interactional space to draw attention to the issues; awareness of the importance of having the issues raised on record as any concerns raised off record do not usually present sufficient grounds for an appeal. In relation to the second situation, the judiciary tend to evaluate sufficient comprehensibility without the examination of the link between the original message and the interpreted one, which means that any additions or omitted parts are not explored [9: 214].

Legal systems thus take an approach that an adequate quality of interpretation is sufficient for the purposes of legal proceedings. But it is important to be transparent about the definitional boundaries of sufficient or adequate interpreting by making linguistically informed decisions. Establishing certification and training programmes for interpreters is crucial, but it should also be accompanied by establishing clear processes for keeping accurate records of original conversations (e.g. interviews, hearings) which are part of the evidentiary stages. Only the combination of these measures can improve the quality of interpreting services, ensure the transparency of proceedings, and enhance access to justice for non-native speakers [9: 211, 44]. Legal systems thus need to aim to introduce these systemic changes as well as raise the legal practitioners’ awareness about the communicative challenges embedded in bilingual proceedings so that the needs of interpreters and non-native speakers could be appropriately accommodated.

## Remote Interpreting in Legal Settings

Given the lack of research on fully online remote hearings, it is important to consider the studies focusing on individual aspects of remote interpreting in different settings. Most of the research on the topic is, however, based on survey data or experimental studies, which means that their applicability and relevance is restricted to general observations or specific contexts. In an experimental study based on pre-set institutional dialogues performed by students, Skaaden [54] reports that turn-taking proves to be a major challenge for remote interpreting as this settings was fairly novel for the research participants. The study highlights the need for raising practitioners’ awareness of the complex skills required for interpreting and the necessity to take precautions to accommodate the interpreters’ needs and enable a smooth communication flow. Similar conclusions were reached by Braun and Taylor [12]. Drawing on the survey results among interpreters and legal practitioners who had prior experience of using video-based interpreting, the authors note that the experiences of participants are varied and depend on many factors, such as circumstances, qualifications, prior experience with an online mode of interpreting. What is important, according to the authors, is to provide specific guidance on how to use videoconference interpreting and remote interpreting as well as how to implement the necessary procedural and communicative adjustments to facilitate interpreting; the recommended adjustments include the installation of technical equipment fit for purposes, introduction of procedural clarity, and implementation of a more inclusive approach to interpreters. Braun and Taylor [12: 81–83] suggest that more exposure to remote interpreting could prepare the participants for the online environment, but highlight that improving general interpreting skills is likely to have most impact on improving the performance of interpreters.

In another part of the research project, the experimental study based on simulated interactions from a script for a police interview, Braun and Taylor [12: 98–99] conclude that videoconference interpreting conducted via a video link to the interview room with a police officer and an interviewee was lower in quality than face-to-face interpreting due to a wide range of problems: linguistic (issues with terminology), discursive (issues with turn-taking and communication management, rapport building), cultural (issues with culture-bound references), and cognitive (overload of cognitive processing and visual information, missing eye contact, earlier onset of fatigue—see also [43]). As a result, the content interpreted via a short-consecutive mode, was not accurate due to omissions, additions and general distortions of meaning. Braun [11] highlights that the cognitive strain experienced by the interpreters during videoconference interpreting reduces their ability to understand the message fully, recognise coherence links or keep track of how feasible their output is. The poor audio/video quality can also present a challenging environment for the work of interpreters. Exploring the interpreter-mediated hearings in magistrates courts in which a prisoner is present via a video link while the rest of the participants, including an interpreter, are in a physical court, Fowler [23] concludes that poor sound quality and restricted coverage of the courtroom and the detainee makes interpreting more difficult and, consequently, deteriorates any pre-existing disadvantages experienced by non-native speakers.

The research on videoconference interpreting or interpreting conducted via a video link draws on the combination of physical and remote environments (i.e. most participants share the physical space and one participant is based remotely), which creates an additional distortion of power relations due to the unequal audio-visual representation of the participants as the positioning of microphones, cameras and monitors defines the extent to which individuals would be seen and heard. Such conditions thus reduce the overall sound quality as well as image and sound synchronisation. The cognitive strain on interpreters or even other participants or difficulties with communication management are thus common problems in such settings. Furthermore, situations when interpreters face multiple images on the monitor (e.g. two images of individual speakers and one shared image with both of speakers in the interview room as seen in [11]) lead to an overflow of visual input and additional challenges, which would not occur in face-to-face interpreting.

Videoconference or video link settings are not comparable to the fully online hearings where all the participants are present remotely and everyone can be seen in a designated space on the monitor, which means that everyone has an equal visual representation and an overview of the participants. The existing videoconference platforms (e.g. Zoom, MS Teams, Webex) are however designed for running online meetings/conferences and are not ideal for court hearings. The HMCTS in England and Wales^7^ is currently rolling out a new Video Hearing service which has been designed in partnership with the judiciary for the purposes of conducting hearings across different jurisdictions (criminal, civil family and tribunal hearings). The service allows the judge to control all the necessary aspects (e.g. (un)muting participants, admitting/dismissing witnesses, pausing/resuming/closing the hearing, consulting lawyers or panel members in private) and includes further features, such as display of clearly visible information on who is participating, use of private meeting rooms while the hearings is paused (participants can administer those independently), video guidance on how to use the service, instructions on how to connect and use the service in several languages. Importantly, the service also enables simultaneous interpreting, although the details about this function have not been released yet. The crucial aspect is that irrespective of the type of platform used, fully online hearings are different from video-mediated hearings in how communication is managed.

## Interpreting in Courtrooms vs. Fully Remote Hearings

This section reflects on the core issues explored in my expert witness report, i.e. the comparison of interpreting in physical court hearings and fully online hearings, leading to the advice on how to safeguard efficient and clear communication processes in online proceedings. In the absence of the research focusing specifically on interpreter-mediated online hearings, my expert witness report drew on the principles of communication management in legal settings, including the formalised and pre-determined turn-taking typical of courtroom/tribunal settings, unequal power relations during hearings [16, 17, 20, 26, 36, 57], and the challenges interpreters face within the communication constraints present in legal settings [9, 34, 55]. Establishing discursive practices and interaction patterns in different modes of hearings before investigating the existing documents related to the relevant communicative practices enabled the report to highlight situations which cause communitive challenges or imbalances in discursive power and propose strategies for addressing potential issues.

During physical court/tribunal hearings, the working conditions are often not suitable as interpreters do not have a designated workspace, which makes it difficult for them to take notes or ensure a smooth communication flow. Interpreters are also reluctant to ask for a table or break as voicing their requests means imposing on the court [34]. The physical settings and acoustics of the room as well as auditory characteristics of speakers impact the general comprehensibility; as a result, there is not usually an ideal place for the interpreter to be seated as there tend to be limitations linked to any location. For instance, interpreters often conduct consecutive interpreting of evidentiary stages (in which their client is examined) and simultaneous interpreting of the rest of the hearing; they are therefore seated next to their client and further away from others so that the consecutive interpreting could be done without obstructing the proceedings. But the inconspicuous presence, the constant refocusing of the attention and turning to face different speakers and recipients (the client, tribunal members, or the lawyer) impacts the interpreter’s ability to hear what is being said by those seated further away and creates an additional comprehension barrier [44]. Furthermore, due to the proximity to their client, the role of the interpreter can be compromised as the clients may share something in confidence or expect support or advice from interpreters [39, 42]. The sitting arrangements also impact the ability of the non-native speaker to complain about the quality of interpreting [42] as this would comprise a face-threatening act towards an interpreter, not to mention the fact that the formalised and pre-determined turn-taking during hearings makes it difficult to interrupt the proceedings to raise any issues. As a result, there are many challenges experienced by interpreters and potentially their clients in physical settings, which can have a negative impact on the quality of interpreting (e.g. lack of optimal working conditions, acoustic deficiencies) or procedural aspects related to interpreting (compromised neutrality of the interpreter-client relationship, reduced opportunities to raise concerns), ultimately leading to the compromised representation of the non-native speakers’ narrative or undermining their ability to follow the proceedings.

Similarly, interpreter-mediated hearings with a video link element, i.e. where only the interpreter is online [e.g. 11] or where the non-native speaker is online (e.g. [22]) while the other participants are in court, pose multiple auditory and cognitive challenges and provide inadequate conditions for interpreters (e.g. several speakers tend to share one microphone and/or microphones are too far and it is often difficult to hear what individuals are saying; the image on the screen and camera coverage may differ for different participants, depending on the seating arrangements; the interpreter may be exposed to the overflow of visual information due to the same images appearing from different angles). Yet, even this type of settings can successfully be used if communication is simplified to one-on-one type of interaction. For instance, in England and Wales, the use of video link is well-established in criminal proceedings with vulnerable victims who sometimes have an intermediary present with them for support with communication. The victim then appears on the screen in court and the communication happens through a question/answer format with one person in court (CPS lawyer, defence lawyer or judge) and the victim (sometimes with the support of an intermediary in the same room) present via a video link, so the pattern of interaction is predominantly one-on-one. Discussing the impact of the evidence provided live via a video link or a pre-recorded video on the deliberation of a mock jury, Ellison and Munro [21] conclude that there does not seem to be much difference between the physical and online modes of evidence presentation when it comes to jury perceptions. Even in interpreted evidence provided via a video link, i.e. conditions much more complex than those tested by Ellison and Munro [21], the premise is that if the turn-taking is managed successfully and procedural clarity is introduced, the interpreting is more likely to be successful [11]. One important aspect to bear in mind is that the video link interpreting is conducted via a consecutive mode, which tends to be more accurate as it allows for more time to make notes and subsequently interpret in coherent chunks [2, 5, 38, 44, 47]: Berk-Seligson [9: 215] reports the accuracy of 70.6% in the consecutive mode as opposed to the simultaneous interpreting where the accuracy is merely 33.3%. This is due to the fact that the objective of the consecutive mode of interpreting is predominantly to summarise the proceedings and there is no time to take notes as the target language output immediately follows the comprehension of the linguistic input. The use of consecutive interpreting is not always preferred though as it slows the pace of the proceedings, so it is often limited to the witness examination of a non-native speaker.

The consecutive mode of interpreting tends to be used in the same way in the fully online hearings offered by the immigration tribunal discussed in the report. The self-represented appellants are thus disadvantaged if the non-evidentiary stages are not interpreted or only summarised for them separately; though the platforms developed specifically for legal purposes (e.g. the Video Hearing services offered by HMCTS) can have an in-built function for simultaneous interpreting. In broader terms, the fully online hearings reduce many of the challenges present in face-to-face settings and video link settings as all participants appear on the same screen, so the visibility within images is comparable and everyone has their own microphone and can adjust the volume of the (in-built) speakers. It is clear who is talking as their image is highlighted or appears in the main frame and everyone has a degree of control over their working conditions (though we cannot ignore inequalities in terms of accessing a representable and calm environment) and can wear a headset to reduce outside noise. One crucial advantage in fully online hearings is that it is arguably easier to indicate a problem or request a break as raising a virtual hand or using the chat function is less obstructive than voicing a request and interrupting the proceedings in the face-to-face environment where it is necessary to wait for a pause in turn-taking. As a result, the fully online settings offers multiple advantages: many communication issues (e.g. broken/delayed speech from one participant) are easier to detect as more participants would notice the issue; there are relatively non-intrusive ways for anyone to raise concerns or request a break; the (perceived) inequalities related to the raised position of the judiciary or tribunal members seating further away from the other participants are less apparent in online settings as everyone appears on the screen in a similar way and everyone can raise their hand or use the chat function; and, finally, the etiquette is simplified in online settings as there is no need for such ceremonies as standing up as the judge enters [27]. The advantages of remote hearings are in the simplification of some communicative and practical aspects (convenience, more control over the working environment, no need for unnecessary proximity between the interpreter and their client). But there are equally some challenges (e.g. building rapport) and some fundamental systemic issues (e.g. access to open justice), which need to be resolved or adapted to.

When preparing the expert witness report, it was important to explore the materials in relation to running online hearings the immigration tribunal was using at the time: the guidance document and the suggested script for starting the online hearings. While the guidance document mainly dealt with the technical aspects, the script addressed many of the potential communication issues in the form of a short briefing at the beginning of the hearings: showing technical issues with a T hand sign; raising a virtual hand or using a chat function to ask a question; request for only one person to speak at a time and remain on mute if not speaking; instructions on how to address technical issues. It is positive to see important communication processes be recognised in the suggested script for conducting online hearings. To improve the awareness and usability of the guidance, cover different circumstances and safeguard the communication management, the report suggested to include the following steps:

- Make the instructions on communication management (i.e. the instructions scripted for the beginning of hearings) available to all participants in advance of the hearing. The same instructions should then be explained for the second time at the beginning of the hearing. The same information will then be presented twice with two different communicative aims: the communicative aim of the written format is to present the crucial information in advance to allow participants enough time to process the information and the aim of the spoken format at the beginning of the hearings is to encourage the participants to use the relevant functions (to voice requests or questions and observe the pre-set communication management rules);
- invite the participants to request a break or voice another request or issue in the least intrusive manner (perhaps chat function so that the tribunal members can address the issue at the most appropriate point);
- introduce a sign for interpreters to indicate the need for the speaker to pause more often (e.g. the interpreter signalling ‘stop’ with their hand). These additional options should help the interpreter or other participants to manage the floor more efficiently;
- encourage the voicing of requests or questions and clarifications of substantive matters or issues with interpreting (through the use of virtual hands or chat functions) to ensure that the participants do not feel like they are imposing;
- make arrangements for an interpreter and appellant to chat informally before the hearing to build rapport and check they can understand each other (e.g. their dialects are mutually comprehensible, there are no audio issues);
- make arrangements for requesting and holding a private lawyer–client consultation with the interpreter;
- clarify arrangements for consecutive & simultaneous interpreting;
- review processes for making decisions on whether cases should be listed for a remote or court-held hearing as self-represented appellants may struggle to make submissions and request a face-to-face hearing;
- in the early pre-hearing stages, elicit any special needs the appellants might have or any other circumstances which could prevent them from active participation in an online hearing.

In sum, there are likely many challenges for interpreters in fully online settings; similarly, there are auditory and psycho-linguistic obstacles for interpreters in a traditional physical hearing format. What is important is that there are considerable advantages that fully online settings offer (e.g. voicing a request, question, clarification in the least obstructive manner) and any potential challenges related to toxic noise or similar disturbances (raised in the expert witness report supporting the judicial review) can be dealt with in an effective manner either via planning the work conditions in advance or addressing the issues as they emerge. Gill [27] shows further advantages related to appellants potentially feeling less exposed or disrespected because the power imbalances are not as apparent in online settings, partially due to simplified etiquette and a more uniform appearance among the participants. The restricted number of attendees in online settings may also lead to the increase in the appellants’ trust in the system and the confidentiality of the process as the risk of their stories being publicly exposed often prevents them from fully participating in the process [27, 28].

Given the health-related measures during the pandemic, it is a reasonable expectation that hearings such as those conducted by the immigration tribunal are delivered via a remote mode. The proposed suggestions, detailed above, should help ensure that all participants in the proceedings feel encouraged to contribute to the efficient communication management. In the physical environment the tribunal members tend to rely on their observation skills to notice if someone is tired or looks like they have a question; the online settings allow for a non-invasive method of voicing requests, which puts the participants on a more equal footing and allows more people to be responsible for raising communication-related issues. Similarly, the existing mechanisms, adopted by the immigration tribunal, to resort to a face-to-face hearing in case of any communication challenges should be sufficient to address potential unfairness or interference with accessing justice (provided that the identification of the appellant’s vulnerabilities related to documented physical or mental health issues is incorporated in the early stages of the process). Procedural clarity on reverting online hearings to physical settings in case of communications issues is crucial: the effectiveness of the hearing relies heavily on the effectiveness of communication and accurate interpretation of the appellant’s narrative. Reducing the challenges can be achieved by encouraging the participants to voice concerns or encouraging interpreters to signal the need for speakers to pause more often; these interventions are arguably easier in online settings than in face-to-face settings.

## Role of Linguistic Expertise in the Case

The unique nature of the case required linguistic expertise to establish the likelihood of challenges for interpreters in fully remote hearings as a pre-emptive prognosis. Although the discussion without the data analysis runs the risk of being speculative, drawing on linguistic principles determining interpreter-mediated communication processes in different settings provides an opportunity to improve discursive practices in legal settings. Originally, before the provision of my expert witness report, the case was revolving around two types of communication-related issues in online settings: (1) narrowly defined challenges for interpreters (i.e. the disproportionate impact from toxic noise impacting comprehension, impoverished linguistic input) and for appellants (reduced credibility when appearing online) as well as (2) individual systemic challenges which could not be resolved within the case (mainly lack of accreditation for court interpreters). Interestingly, the case required expertise in several areas of forensic linguistics, namely courtroom discourse, narrativisation practices in immigration tribunals, discursive practices embedded in legal proceedings, role of interpreters in legal settings, legal-lay procedural challenges, record keeping practices, digitisation of the justice system, and research around deception detection and credibility assessment.

Drawing on several areas of expertise lead to a structured approach to addressing the points raised in the letter of instructions, paying attention to different communicative contexts (access to lawyers in online settings, access to courts for those without a lawyer), focusing not only on interpreters but also on appellants and the support they can access (e.g. access to technology and quiet space for appellants and interpreters), and suggesting further improvements for voicing requests in online settings. This required conducting investigations which lay outside the issues originally defined in the letter of instructions.^8^ The other reason for conducting independent investigations was to reduce the impact of potential contextual bias [37], which is more likely to be displayed when responding to the existing expert witness report rather than being part of the initial evidentiary stage. As a result of the wider scope of investigations, the report did not focus on interpreters only, but on the broader context in which interpreting happens and the remedial strategies for establishing smooth communication flow.

In addition to advising the court on the linguistic principles guiding online and face-to-face settings, it was equally important to refute common misconceptions, such as the role of perceptions in credibility assessment. One of the misplaced concerns on the agenda was related to the notion that it is difficult to read people’s reactions in online settings (cf the Nuffield Foundation report on remote hearing in family courts^9^), which can negatively impact the tribunal members’ ability to assess credibility. From research studies focusing specifically on credibility assessment [7, 45, 53, 58, 59] we know that linguistic cues related to internal consistency and external consistency are much more reliable and more objective indicators of credibility. Given that the document on credibility indicators used by the immigration tribunal clearly stated that the assessment should not be based on subjective assumptions and preconceptions, it was necessary to highlight that the attempts to assess body language or demeanour should not form part of credibility assessment in any setting, online or face-to-face.

In relation to the wider expertise in forensic linguistics necessary for the case, it is equally important to note that the court, essentially, had two reports from the experts with complementing expertise: translation studies (from the expert witness for the appellant) and spoken interaction in legal settings. Giving an opportunity for both expert witnesses to narrow down contentious points would have been useful (e.g. such process is part of procedural rules in relation to experts and assessors in England and Wales^10^), but this is rarely part of adversarial proceedings. Another concerning aspect was that upon requesting access to observe online hearings, the tribunal initially arranged to do so but then cancelled the request with the explanation that it would not be procedurally fair if only one expert had access to the observations. This raises concerns from the point of view of procedural justice (both experts could have been offered observations) as well as open justice (it was not possible to observe online hearings independently of the case).

Despite the fact that the case did not involve data analysis, it presented a unique opportunity to provide the rationale for two crucial systematic changes (accreditation programme for court interpreters, audio/video recording of evidential stages and hearings), reflect on the existing communicative processes and propose methods for safeguarding the interactive procedures and transparent communicative processes. The case also illustrates that linguistic expertise can be part of the planning, implementation and quality assurance processes across a wide range and format of legal proceedings.

## Summary

As many jurisdictions across the world have adopted or are in the process of adopting remote hearings as a standard provision within the respective justice systems (e.g. Civil Rules of Procedure in Ohio^11^ are being updated to ensure more clarity around online proceedings), the linguistic principles embedded in communicative practices can help us understand the effect of different modes of interaction on the participants and should thus be considered within procedural rules. As the analysed case shows, the practitioners’ reactions to remote hearings have been mixed, with some expressing concerns as to the confidentiality of the proceedings, credibility perceptions, effect on lawyer-client communication, barriers related to digital literacy, and access to justice for the vulnerable, to name a few [6, 14]. Nevertheless, as practitioners gain more experience with the remote environment and as user experience is embedded into the platform design, the overall reactions to online hearings tend to be more positive. Byrom et al. [14] report that 71.5% of legal representatives who participated in remote hearings were broadly happy with their experience, though it is important to note that the success of online hearings tended to depend on such aspects as the agreement of both parties in respect to the outcome, absence of technical issues, participation in a video hearing rather than a telephone hearing, and prior experience with remote hearings. The report recommends using the remote mode for hearings which are likely to be less contested, rely on interlocutory focus and have both parties represented by a lawyer. It is crucial to further research the impact of remote hearings on access to justice. For this to happen, the courts need to address the concerns in relation to open justice and practical limitations of accessing remote hearings as access details can be difficult to obtain (login details seem to be more readily available for journalists rather than the wider public, causing limitations to the principle of open justice, see [14]).

Until further research into fully online hearings emerges, interpreter-mediated communication can be enhanced if more participants are enabled to have their needs and concerns expressed in the most unobstructive yet clear way. As the article shows, there are multiple challenges in such culturally and linguistically diverse communicative environment as immigration proceedings. Socio-legal and ethnographic research [1, 3, 27, 29, 51] as well as linguistic research [9, 32, 48, 57] show that the procedures are flawed with multiple communicative issues: narrativisation disjunction, chaotic interactional dynamics, imbalanced power relations, the communicative culture of mistrust, incompleteness of records from evidentiary stages. The fact that communication in immigration proceedings is often interpreter-mediated creates an additional factor which needs to be accounted for in terms of turn-taking management, participant dynamics, and accuracy of the narrative presentation. The challenges for interpreters and appellants are thus manifold and need to be managed carefully by legal professionals.

By drawing on linguistic principles governing communication management and discursive practices, the expert witness report aimed to present challenges and advantages embedded in interpreting practices within face-to-face settings and fully online hearings, refute common misconceptions on assessing credibility, propose appropriate safeguarding measures, and encourage all parties to raise concerns and contribute to the proceedings. Improving the accuracy of interpreting in online settings is a complex question and incorporates improvements in relation to the design of the software platforms, digital literacy, experience with online proceedings, procedural adaptations, and above all training and enhancing of interpreting skills [11].

The additional layer of issues to be addressed is related to systemic aspects: the accreditation of legal interpreters and the implementation of audio/video recording of the proceedings. The recording is crucial to ensure all multi/bilingual original interactions, including the parts of proceedings which are interpreted via a simultaneous mode, are reflected in an accurate and cost-efficient manner (see [39] for the practices of adversarial interpreting involving an additional interpreter to monitor the interpreting process). Fully online proceedings are arguably easier to record than those held in court due to the position of microphones in the courtroom/tribunal. It is equally important to consider the risk of parties secretly recording the proceedings. Although covert recordings are prohibited, it can be difficult to prevent parties from making private recordings during remote hearings; as unlawfully obtained evidence may be admissible in practice if other sources are not available [52], it is better to have official recordings in case of appeals. Another strong argument in favour of recordings is that they can be used not only to assess the performance of interpreters for the purposes of appeals but also for research purposes and long-term improvement of interpreter-mediated communication practices in multilingual legal settings. The appropriate standards for the qualifications of court interpreters, regular training programmes and keeping audio recordings are thus essential pillars on which the professionalisation of the services and access to justice for non-native speakers should be based. In sum, the requirement for linguistic expertise in the discussed judicial review case illustrates how applied linguistics can contribute to the evaluation and enhancement of discursive practices as well as planning of preventive and quality-assurance systemic measures.

## Data Availability

There is no data.

## Footnotes

1 https://remotecourts.org/.

2 https://insidehmcts.blog.gov.uk/2021/06/09/remote-hearings-their-role-in-extending-access-to-justice/.

3 https://www.altalex.com/documents/news/2022/01/31/smart-court-chat-bot-e-fascicoli-personali-consultabili-online-la-giustizia-viaggia-su-app.

4 https://www.reuters.com/legal/litigation/jones-day-wins-bid-opposing-all-virtual-trial-sutter-antitrust-case-2022-01-18/.

5 https://commonwealthmagazine.org/courts/can-a-parents-right-to-their-child-be-terminated-via-zoom/.

6 https://www.kstcr.cz/cz/jak-se-stat-soudnim-tlumocnikem.

7 https://www.gov.uk/government/news/hmcts-presents-the-5th-annual-public-user-event.

8 It is important to note that the scope of investigations was limited as the deadline for the report submission was only 2 days after the receipt of the letter of instructions.

9 https://www.nuffieldfjo.org.uk/wp-content/uploads/2021/05/nfjo_remote_hearings_20200507-2-.pdf.

10 Practice Direction 35 https://www.justice.gov.uk/courts/procedure-rules/civil/rules/part35/pd_part35.

11 https://www.jdsupra.com/legalnews/modernizing-ohio-s-civil-rules-of-9517055/.

## Code Availability

All software used for the analysis is cited in the article.
